# Editorial: Exploring oral microbiota dysbiosis as a risk factor for oral and non-communicable diseases

**DOI:** 10.3389/froh.2025.1611120

**Published:** 2025-04-25

**Authors:** Denis Bourgeois, Giovanna Orsini, Florence Carrouel

**Affiliations:** ^1^ Laboratory “Health, Systemic, Process” (P2S), UR4129, University Claude Bernard Lyon 1, University of Lyon, Lyon, France; ^2^ Department of Clinical Sciences and Stomatology (DISCO), Università Politecnica Delle Marche, Ancona, Italy

**Keywords:** oral health, microbiota, non-communicable diseases, public health, symbiosis

**Editorial on the Research Topic**
Exploring oral microbiota dysbiosis as a risk factor for oral and non-communicable diseases

The human oral cavity harbors one of the most diverse microbial communities, consisting of hundreds of bacterial species that co-exist in a finely tuned ecological balance. This intricate ecosystem, collectively referred to as the oral microbiota, plays a pivotal role not only in maintaining oral homeostasis but also in modulating systemic health through complex host–microbe and microbe–microbe interactions (1). However, when this balance is disrupted—a condition termed oral microbiota dysbiosis—a shift occurs from a symbiotic to a pathogenic microbial profile. This imbalance is increasingly implicated in the initiation and progression of a wide array of diseases, extending far beyond the oral cavity. Emerging evidence suggests that oral dysbiosis acts as a biological driver and risk modifier for numerous non-communicable diseases (NCDs), including cardiovascular, respiratory, neurodegenerative, and metabolic disorders (2). These links underscore the oral cavity's role as a critical interface between environmental exposures and systemic pathophysiology. This editorial introduces a collection of studies that collectively explore the multifactorial nature of oral microbiota dysbiosis, elucidating its mechanistic underpinnings, clinical implications, and potential as a target for novel diagnostic and therapeutic strategies.

Dysbiosis of the dental biofilm is associated with common oral diseases such as gingivitis and periodontitis. Carrouel et al. investigated interdental microbiota in first-trimester pregnant women with clinically healthy periodontium, highlighting the importance of monitoring early gingival conditions during pregnancy—a time of increased susceptibility to inflammation and dysbiosis (Carrouel et al.). The pathogenic role of structured oral biofilms, composed of bacteria within a protective extracellular matrix, is critical to disease persistence. In this context, Chen et al. demonstrated how a stannous-containing sodium fluoride dentifrice may modulate microbial composition and inhibit pathogenic interactions, offering a preventive avenue against dysbiosis-related damage (Chen et al.). In parallel, Bourgeois called for preventive strategies centered on modulating the oral microbiota, underlining its foundational role in oral health (Bourgeois).

The implications of dysbiosis extend beyond oral diseases. A study by He et al. employing two-sample Mendelian randomization in an East Asian population uncovered potential causal links between the oral microbiome and five respiratory infections, including tonsillitis, chronic sinusitis, bronchiectasis, bronchitis, and pneumonia (He et al.). These findings underscore the anatomical continuity between the oral cavity and respiratory tract, underscoring the systemic influence of microbial shifts in the oral niche.

Pathobionts such as *Porphyromonas gingivalis*, have emerged as central players in systemic disease pathways. Wu et al. reviewed the roles of *P. gingivalis* outer membrane vesicles (OMVs), emphasizing their ability to transport virulence factors to distant tissues, modulate host immune responses, and contribute to diseases including cardiovascular disease, Alzheimer's disease, respiratory disorders, and cancer (Wu et al.).

In pediatric populations, Lin et al. examined microbial transitions from caries to apical periodontitis in children with severe early childhood caries, highlighting dysbiosis as a driver of disease progression (Lin et al.). Complementing this, Boisen et al. explored oral biofilm phenotypes in caries-active vs. caries-free children, identifying microbial profiles that could inform risk stratification and early interventions (Boisen et al.). Muhammad et al. drew attention to the less-explored connection between oral microbiota and childhood stunting, proposing mechanisms by which dysbiosis may impact nutrient absorption and immune regulation (Muhammad et al.).

Further microbial characterization by Carrouel et al. utilized Socransky's complex classification to identify periodontopathogens in pregnant women, reporting a predominance of the orange complex and emphasizing the role of interdental hygiene in mitigating dysbiosis (Carrouel et al.). In a metatranscriptomic analysis, Ovsepian et al. identified differentially expressed genes and virulence factors associated with periodontitis, offering insights into the functional disruption characteristic of dysbiotic states (Ovsepian et al.).

Environmental and lifestyle factors also shape the oral microbiome. Senaratne et al. demonstrated the impact of waterpipe smoking on the salivary microbiota, illustrating how exogenous exposures can disturb microbial equilibrium and potentially elevate disease risk (Senaratne et al.).

Collectively, these studies emphasize the intricate and systemic influence of oral microbiota dysbiosis, underscoring its role as a modifiable risk factor not only for localized oral conditions such as periodontitis and dental caries, but also for a growing list of systemic NCDs (3). This growing body of evidence shifts the paradigm from treatment to prevention—positioning oral health as an essential component of general health strategies (4). Crucially, oral dysbiosis is not inevitable; it is shaped by modifiable lifestyle factors, including oral hygiene practices, dietary habits, and environmental exposures.

Preventive measures grounded in good oral hygiene—such as effective interdental cleaning, fluoride use, and biofilm control—can significantly reduce the risk of dysbiosis and its sequelae (Bourgeois). However, prevention must also address upstream determinants. Nutritional interventions, particularly those aligned with the principles of the Planetary Health Diet, offer a promising avenue for modulating the oral microbiome (5). Diets rich in fiber, polyphenols, and unsaturated fats while low in refined sugars and processed foods have been shown to support a symbiotic oral microbiome, reduce systemic inflammation, and promote overall metabolic health. Furthermore, these diets benefit planetary sustainability, making them an ideal nexus between individual and ecological health.

A deeper understanding of host–microbiome interactions and microbial ecology is therefore essential not only for managing disease but for advancing integrated, microbiome-informed preventive strategies. Future research should prioritize translating these insights into personalized interventions—ranging from tailored oral hygiene protocols to microbiome-targeted therapeutics and nutrition-based strategies. In doing so, we can shift from reactive treatment to proactive preservation of oral symbiosis and systemic resilience, contributing to healthier individuals and healthier planet.
